# Turkish Adaptation of the Positive–Negative Relationship Quality (PN-RQ) Scale: A Reliability and Validity Study

**DOI:** 10.3390/bs9090100

**Published:** 2019-09-15

**Authors:** Arzu Araz, Duygu Güngör, Eda Aşçı

**Affiliations:** Department of Psychology, Dokuz Eylül University, 35390 Izmir, Turkey; duygu.gungor@deu.edu.tr (D.G.); eda.asci@ogr.deu.edu.tr (E.A.)

**Keywords:** positive–negative relationship quality, reliability, validity, confirmatory factor analysis (CFA), bifactor

## Abstract

Background: The present study investigates the reliability and validity of the Positive–Negative Relationship Quality (PN-RQ) scale in Turkey. This study aims to test different factorial models including orthogonal factors model, correlated factors model, one-factor model, and bifactor model. Methods: In order to determine the validity and reliability of the scale, two studies were performed. The first was carried out with emerging adults (university students) who were in a romantic relationship (148 females, 43 males, and 2 unknown) and had the main purpose to examine the structure validity of the measurement tool in the Turkish sample with an exploratory analysis. Study 2 was performed online with 513 married adults (359 females, 149 males, and 5 unknown); confirmatory findings and criterion validity studies were added. Results: Exploratory factor analyses revealed that relationship quality had a two-factor structure and that there was also a negative relationship between the factors. Confirmatory factor analyses on the married sample showed that the bi-factor model provided evidence for the multidimensional nature of the scale. Both studies demonstrated high internal consistency. Conclusion: There is evidence for reliability and validity in the Turkish version of the PN-RQ scale to measure both positive and negative aspects of the relationship. The PN-RQ scale will be highly functional for social and clinical psychologists who work on close relationship issues in Turkey.

## 1. Introduction

Relationship quality is the most frequently studied construct in close relationships [1]. Following the initial studies by Hamilton in 1929 and Terman in 1938, a large number of studies with respect to relationship quality have been carried out in order to identify relationship quality, its determinants and consequences [2]. Relationship quality is not only investigated by psychologists, but also by researchers from the field of sociology, family studies, and other disciplines [3,4].

Relationship quality involves individuals in a wide range of relationships, including those who live together, who are married, and who have a romantic relationship. However, due to its legal association, it is easier to identify married couples, which is why most relationship quality studies are carried out with them [5]. In addition to this, there are studies on relationship quality between married and unmarried couples. In a recent study [6] examining the relationship quality of cohabiting and married couples, direct married couples’ relationship quality was reported highest, whereas it was reported the lowest with couples who cohabit and do not plan to marry. Marrieds who premaritally cohabited and cohabiters with plans to marry remained undifferentiated and indicated an intermediate level of relationship quality.

Relationship quality is a determinant of quality of life and well-being [7,8]. In a meta-analysis conducted with 126 articles and more than 72,000 participants, examining the relationship between marital quality and physical health revealed that high marital quality was associated with better health and lower mortality rate [9]. In order to determine whether physical and mental health associated with marriage also applied to college students, a study with 1621 dating college students was conducted [10]. It was found that individuals in committed relationships were less likely to be overweight and less likely to experience mental health problems. But there were no significant differences between single students and students in romantic relationships. Moreover, relationship quality has proven to have several effects on children [11].

According to Fletcher et al. [12], for researchers who want to measure relationship quality it is very challenging to decide which scale to use, as there are many measurement tools and the terms used to define the measured subject differ. Some of the self-report measurement tools used to measure relationship quality are as follows: Marital Adjustment Test [13], the Dyadic Adjustment Scale [14], the Quality of Marriage Index [15], the Kansas Marital Satisfaction Scale [16], the Golombock–Rust Inventory of Marital State [17], the Relationship Assessment Scale [18], the Perceived Relationship Quality Components [12], and Relationship Quality Scale [19].

The quality of relationship is studied by using several terms such as “satisfaction, adjustment, happiness, companionship, success” [3,4,20]. For this reason, there is still no consensus on how these terms will be defined or measured [2]. According to Fincham and Rogge [3], researchers who work on marriage start from two basic approaches. First, the *relationship* or *interpersonal approaches* typically focus on interaction patterns such as companionship, communication, or conflict and is preferably called adjustment. Second, the *intrapersonal approach* involves the couples’ subjective evaluations on marriage and works with terms such as marital satisfaction or marital happiness. Even though the authors find both approaches valuable, they have criticized them for their problems with measurements. According to Fincham and Rogge [3], researchers have worked on the same subject despite the conceptual distinctions they made when introducing their research. For instance, The Marital Adjustment Test and the Dyadic Adjustment Scale, which are the two most commonly used measures of relationship quality, include items that evaluate both intrapersonal and interpersonal processes. In sum, relationship quality is traditionally conceptualized as unidimensional, and a total score is obtained. According to Fincham and Rogge [3], evaluating relationship quality unidimensionally blurs this important phenomenon and over-simplifies the theories.

Measurements of relationship quality with the spouse or the romantic partner are important both in understanding the nature of the relationships and in arranging the intervention programs that will help to make the relationships more positive. Self-report scales that have proven reliability and validity in determining relationship quality are essential measurement tools for researchers. In Turkey, some of these tools are used to examine marital or dating relationships (e.g., Dyadic Adjustment Scale, Marital Adjustment Test). Even if the relevant measurement tools provide valuable information on relationships in Turkey, in all the cases, relationships are regarded as unidimensional. However, as mentioned above, relationships have both positive and negative qualities at the same time, in other words, they are bidimensional.

The aim of this research is to adapt the Positive–Negative Relationship Quality scale [21], which measures the quality of relations in two dimensions, into Turkish. Additionally, this study aims to test different factorial models including orthogonal factors model, correlated factors model, one-factor model, and bifactor model. Bifactor model is especially important to see whether the relationship quality can be approved as a two-dimensional structure by different latent variables rather than as proposed by Rogge et al. with item-response theory.

In order to determine the validity and reliability of the scale, two studies were performed. The first was carried out with a student sample and had the main purpose to examine the construct validity of the measurement tool in the Turkish sample with an exploratory analysis. In the second study, confirmatory findings and criterion validity studies were added. Analyses in both studies were performed using IBM SPSS statistics 23 (IBM Corp. Released 2015 IBM SPSS Statistics for Windows, Version 23.0. Armonk, NY, USA) and Lisrel 8.80 (Scientific Software International, Inc. Released 2006 Lisrel for Windows, Version 8.80. Lincolnwood, IL, USA).

## 2. Study 1

### 2.1. Methods

#### 2.1.1. Participants

The sample consisted of 193 university students from different academic years. Inclusion criterion for the study was being in a romantic relationship. The gender distribution was uneven, comprising 76.7% females and 22.3% males, while 1% did not state their gender at all. The respondents’ mean age was 21.63 years (SD = 2.36). The average length of their relationships was about two years, ranging from two weeks to nine years (SD = 23.19, measured in months).

#### 2.1.2. Instruments

Within the scope of the first study, the answers to the Positive–Negative Relationship Quality (PN-RQ) scale as well as information about gender, age, department, and duration of the relationship from the participants via demographic information questionnaire were obtained.

*Positive*–*Negative Relationship Quality Scale.* The PN-RQ scale was developed by Rogge, Fincham, Crasta, and Maniaci [21]. The starting point in this scale is that people who have romantic relationships have both positive and negative feelings towards their partners, and that these feelings are partially independent of each other. According to the researchers, relationship quality is two-dimensional, and positive qualities of relationship are distinct from negative qualities. In addition, relationship quality and relationship satisfaction are considered as synonymous terms.

During the development process of the scale, the researchers carried out three studies. The first study consisted of 1814 emerging adults (77% female), while the second study was accomplished with 787 participants (66% female) who were at least 18 years old and had a romantic relationship. In the second study, which was performed online, a follow-up study was done with 473 participants after two weeks. The third study was conducted as an intervention study, with 74 (69% female) pretest and post-test measures.

As a result, the researchers found (a) that positive and negative qualities were discrete dimensions through a confirmatory factor analysis, (b) that the two dimensions were adjusted through optimized measurement of the item response theory, (c) that the differences between the indifferent (perception of both positive and negative qualities on a low level) and ambivalent (perception of both positive and negative qualities on a high level) relationships that unidimensional scales are not able to reveal, can be determined through PN-RQ, (d) that PN-RQ scales are highly responsive to a change by time, (e) that the predictive validity of the PN-RQ scale scores is stronger than that of the unidimensional scale, and (f) that unique longitudinal information is obtained using a two-dimensional PN-RQ scale in the intervention. Researchers have argued that PN-RQ is highly promising to provide development in both basic and applied research when they evaluated the results as a whole.

PN-RQ scale measures both the positive and negative aspects of the relationship, and there are both long and short version of the scale. The long version of the scale contains eight items for each dimension. The long version is recommended due to necessitated precision and power when working with small samples. In the short version, there are four items for each dimension, and it has been developed for measurement tool sets that are more laborious, such as diary studies or telephone surveys.

The positive subscale of PN-RQ measures the positive characteristics of a person’s relationship. The participant is asked to consider his/her relationship with his/her partner only by considering the positive qualities of the relationship and ignoring the negative qualities. In the long version of the scale, the adjectives enjoyable, pleasant, strong, alive, fun, full, energizing, and exciting are used in this order, while in the short version only the first four adjectives are used. Higher scores indicate greater positive relationship quality. The negative subscale of the PN-RQ, on the other hand, measures the negative qualities of a person’s relationship. The participant is asked to evaluate his/her relationship with his/her partner by considering only the negative qualities of the relationship and ignoring the positive qualities. In the long version of the scale, the adjectives miserable, bad, empty, lifeless, unpleasant, dull, weak, and discouraging are used in this order, while in the short version only the first four adjectives are used. Higher scores indicate greater negative relationship quality. The participant evaluates all items on a 6-point rating system (not at all = 0, a little true = 1, somewhat true = 2, mostly true = 3, very true = 4, completely true = 5). Cronbach’s alphas for both the positive and the negative subscales in the first study were 0.95, in the second study for the long version of the positive subscale 0.96, and for the short version 0.94. In the third study, Cronbach’s alphas for the positive subscale were 0.94, and for the negative subscale 0.84.

In the phase of adaptation of the Positive–Negative Relationship Quality scale, the scale items were initially translated into Turkish by three researchers of the Psychology Department who are proficient in English; the translations by the researchers were found to be substantially consistent with each other. For a few items whose translations differed, the evaluations of two more judges from the field of psychology were considered to complete the scale. In the final stage, another researcher in psychology with a degree of English proficiency back-translated the items. It resulted that the translation corresponded to the original scale.

Prior to the actual application, a preliminary study was conducted with 99 students. They were asked to evaluate which one of the expressions “mostly true” or “very true” they would rank in priority on a scale ranging from “not at all true” to “completely true”. Ultimately, the majority decided that the expression “mostly true” was less true than the expression “very true”, which was accordingly applied to the Turkish writing. Taking into account Turkey’s cultural features and the Turkish language during the process of the Turkish adaptation of the PN-RQ scale, while prior to positive and negative adjectives for unmarried participants who had a relationship the expression “my relationship with my romantic partner” was used.

#### 2.1.3. Procedure

Data were collected at three Turkish universities in the Aegean region in Turkey by researchers themselves. The application was carried out in a class environment. The questionnaire sought information about demographic variables at the beginning, followed by the PN-RQ. All participants were informed in writing that participation was voluntary and anonymous, and that their responses were confidential. All study procedures were reviewed and approved by the Ethics Committee of the Dokuz Eylul University on the 10 January 2018, and all participants provided informed consent.

### 2.2. Results

#### 2.2.1. Structure Validity and Reliability

The exploratory factor analysis was run with the principal axis factoring extraction method. As can be seen in Table 1, two factors with Eigen values higher than one were extracted. The first factor—can be named as a *positive quality factor*—explained 50.66% of the variance, and the second factor (*negative quality factor*) explained 10.96% of the variance. In the 8-item scale, the Cronbach’s alpha for the positive factor was 0.93 and for the negative factor 0.90.

#### 2.2.2. Correlation of PN-RQ Scales

Correlation between PN-RQ positive and negative subscales was r = −0.63, yielded that they share 40% of their variance. Correlations with this one-item measurement and PN-RQ positive and negative scales were r = 0.70 and r = −0.59, respectively.

## 3. Study 2

### 3.1. Methods

#### 3.1.1. Participants

The sample consisted of 513 married adults (70% females; 29% males; 1% with no indication). The respondents mean age was 41.73 (SD = 10.46), and age ranged from 22 to 66 years. The average length of their marital relationships was 14.32 years (SD = 11.34), while the average length of dating before marriage was 3.79 years (SD = 3.44). Before their marriage, 449 respondents indicated that they have been involved in a dating relationship with their marital partner, whereas 59 participants had an arranged marriage. Seventy-three percent indicated that they had children, while 27% indicated that they had none. The majority with 256 participants reported having a college degree, 135 participants a postgraduate degree, 52 participants a high school degree, five participants a secondary school degree, and only three participants a primary school degree. Three participants did not respond to negative adjectives, but were not excluded from the analysis.

#### 3.1.2. Instruments

In this study, participants were given a PN-RQ scale, a Dyadic Adjustment Scale (DAS), and a demographic information questionnaire. Different than in the first study, the expression that was used prior to positive and negative adjectives for married participants was “my relationship with my spouse”.

*Dyadic Adjustment Scale.* The scale developed by Spanier [14], contains 32 items and aims to evaluate relationship quality of married or cohabiting couples. It consists of four subscales: dyadic satisfaction, dyadic cohesion, dyadic consensus, and affectional expression. The total score of the scale consists of the sum of all items and ranges from 0 to 151. Higher scores indicate an increase in relationship quality. The Turkish version of the scale was created by Fışıloğlu and Demir [22]; its Cronbach’s alpha internal consistency coefficient was 0.92, the split-half reliability coefficient was 0.86, and the correlation with the Marital Adjustment Scale by Locke and Wallace [13] was 0.82. In this study, Cronbach’s alpha was found to be 0.93.

Information about gender, age, level of education, duration of marriage, and whether the participants had children or not were acquired through the demographic information questionnaire.

#### 3.1.3. Procedure

Recruitment of married participants was accomplished by means of social media. Participants were given access to a secure web link, which referred them to a reliable online survey system (Qualtrics). An informed consent was provided on the first page of the questionnaire, which explained the aim of the study and contact details of the head researcher. Questions containing demographic information were followed by the PN-RQ scale, and finally by the DAS. After completion of the survey, participants were briefly thanked.

### 3.2. Results

#### 3.2.1. Structure Validity and Reliability

An exploratory factor analysis was run with a principal axis factoring extraction method. Two factors with Eigen values higher than one were extracted (see Table 2). The first factor—can be named as a *positive quality* factor—explained 65% of the variance, and the second factor (*negative quality factor*) explained 13% of the variance. In the 8-item scale, the Cronbach’s alpha for the positive factor was 0.96 and for the negative factor 0.97.

#### 3.2.2. Confirmatory Factor Analysis

**Figure 1:**
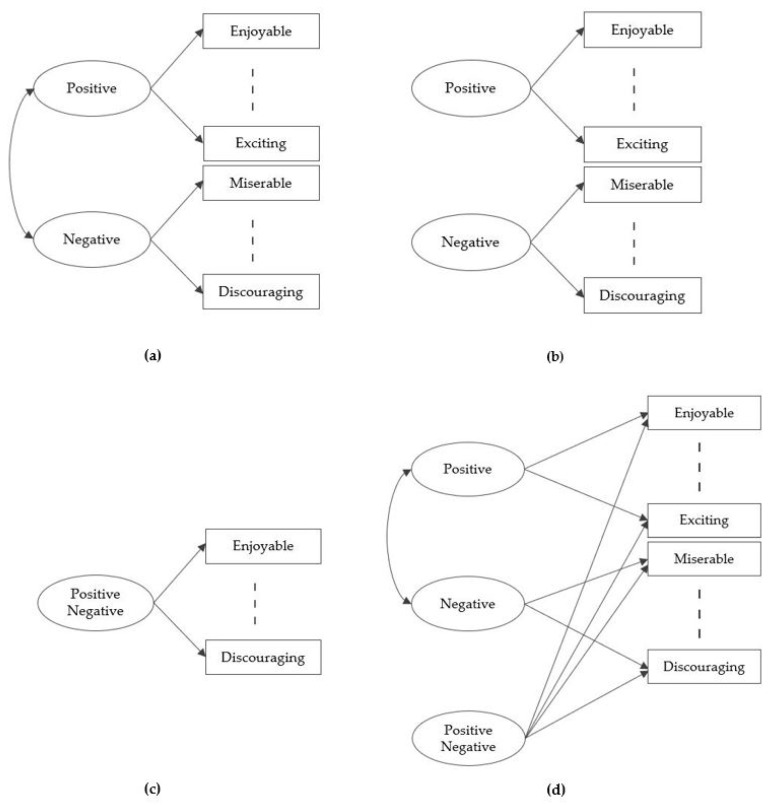
(**a**) Model 1: Correlated two-factor; (**b**) Model 2: Orthogonal two-factor; (**c**) Model 3: One-factor; (**d**) Model 4: Bifactor.

In order to test the multidimensional structure of the PN-RQ scale, as can be seen in Figure 1, a two-factor model, in which the positive and the negative factors are related (Model 1), a two-factor model, in which the positive and the negative factors are orthogonal (Model 2), a single-factor model (Model 3), and a bifactor model (Model 4) were tested. Conceptual models are represented in Table 3. LISREL 8.80 statistical package program was used to test alternative models. Normed chi square (χ^2^/*df*), RMSEA (root mean square error of approximation), CFI (comparative fit index), and NFI (normed-fit index) values are reported. The chi-square value is highly sensitive to the sample size, so although it is reported in almost all studies, other fit indices are more useful [23]. Browne and Cudeck [24] pointed out that practical experience made researchers subjectively use the value of 0.05 or less for a proof of close fit. However, they proposed 0.08 or less for reasonable error. They also highlighted that models with RMSEA value greater than 0.1 might not want to be employed. According to Thompson [25], CFI and NFI values should be greater than 0.95 for acceptable fit.

**Figure 2:**
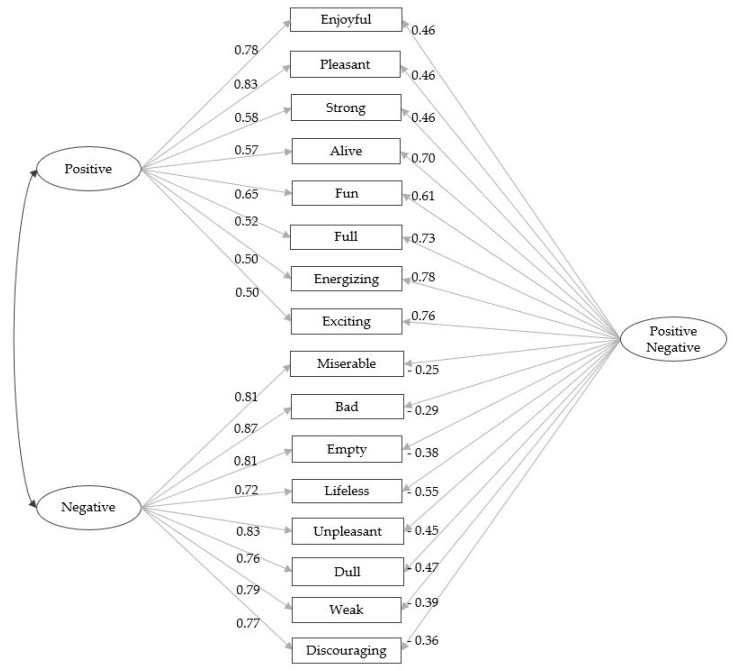
Bifactor Model.

The best model among these four has been determined as the bifactor model. Estimated parameters of the bifactor model are presented in Figure 2.

#### 3.2.3. Correlation of PN-RQ Scales

The correlation between PN-RQ positive and negative subscales was r = −0.64 and yields that they share 41% of their variance, which is higher than in the original study. Moreover, PN-RQ positive and negative subscales were highly correlated with DAS, r = 0.72 and r = −0.70, respectively.

#### 3.2.4. Bivariate Distinctiveness

To investigate the advantage of using a bivariate relationship scale, median splits were created using the 8-item version of the scale for the subgroups as in the original study. The median of positive qualities’ factor was 33 and the median of negative qualities’ factor was 12 in this sample. Then, a univariate analysis of variance was run to investigate differences in these four groups on their measure of relationship satisfaction based on the Dyadic Adjustment Scale (see Table 4).

According to the results of the analysis of variance, a statistically significant difference was found among the groups. To identify among which groups differences occurred, post hoc analyses using the Scheffé post hoc criterion for significance revealed that the satisfied group had the highest satisfaction scores, whereas the dissatisfied group had the lowest. However, ambivalent and indifferent groups did not differ in their satisfaction scores (see Table 4).

#### 3.2.5. Short Version CFA

In order to determine the best short version of the scale, an alternative model strategy within the structural equation modeling was followed. The bifactor model was tested with the first four adjectives in the original scale (χ2/df = 4.37; RMSEA = 0.082; CFI = 0.99; NFI = 0.99), with the last four adjectives (χ2/df = 2.45; RMSEA = 0.054; CFI = 1; NFI = 1) and with the adjectives that had the highest factor loading in this study (χ2/df = 2; RMSEA = 0.046; CFI =1; NFI = 1). It was found that the best short version was the adjectives with the highest factor loading in the exploratory factor analysis (for the positive subscale, items include fun, full, energizing, exciting, and miserable, for the negative subscale, items include bad, empty, and unpleasant). In this 4-item scale, the Cronbach’s alpha for the positive factor was 0.95, and for the negative factor it was also 0.95.

## 4. Discussion

The aim of this study was to adapt the PN-RQ scale into Turkish and to determine the validity and reliability of the scale. Exploratory factor analysis results, obtained from both the first and the second study, revealed that the relationship quality can be regarded as a bifactorial structure and that there is also a negative relationship between these factors. For the criterion-related validity of the correlation of PN-RQ with DAS, which is frequently used in the literature, the Cronbach’s alpha internal consistency coefficients, obtained in both the first and second studies, provide evidence for the reliability. All of the findings in the Turkish sample are in line with the findings obtained in the original scale study.

In addition to the findings of the exploratory factor analysis, the identification of the bifactor model, as the best model among the various confirmatory factor models that were tested using the married sample, provides evidence for the multidimensional nature of the PN-RQ scale. The bifactor model has been operated with particularly with growing interest in the analysis of the dimensionality problem in psychological research. Bifactor models may be practical when there is a general factor ensuring commonality among domains that are item-related, when there are multiple domain or group factors which go beyond the general factor and influence the particular domain, or when both domain factors and the common factor are relevant for the research [26].

In this study, the bifactor model was investigated with one positive quality factor using positive adjectives as indicators, one negative quality factor using negative adjectives as indicators, and one global factor that was indicated by both positive and negative adjectives. This global factor allowed the model to take into account participants with high scores in positive and negative factors simultaneously, named as the ambivalent group, as well as participants with low scores, the indifferent group.

As mentioned above, the relationship quality is conceptualized as one-dimensional in the literature, and a total score is obtained by means of self-report instruments. According to Fincham and Rogge [3], relationship quality is two-dimensional and the positive qualities of the relationship are distinct from their negative qualities. One-dimensional evaluation of the relationship quality blurs this important phenomenon and over-simplifies theories. Based on these criticisms, the PN-RQ scale developed by Rogge and colleagues [21] measures relationship quality considering its both positive and negative aspects. It also makes it possible to categorize people into four different groups: satisfied (who rate positive adjectives with high scores and negative adjectives with low scores), dissatisfied (who rate negative adjectives with high scores and positive adjectives with low scores), indifferent (who rate both positive and negative adjectives with low scores), and ambivalent (who rate both positive and negative adjectives with high scores). Comparisons that can be made after such a categorization will be much more distinct than the comparisons that are made based on a single total score.

Both the long and the short version of the PN-RQ scale are highly functional for researchers. The short version is suitable for those who will work with small sample groups, and the long version for those who will use a large number of measurement tools. The long version of the scale is already conveniently short.

The above-mentioned strengths have some limitations. In this study, the test–retest reliability of the scale was not investigated. It has been studied with university students who already had a relationship or married people. In the first and second study, the sample consisted mostly of female participants (76% and 70%, respectively). Moreover, in the first study, only heterosexual students participated, and a significant difference can be detected in the length of relationship in both of the studies. It would be insightful to have considered the difference within this variable both qualitatively and quantitatively. Future studies that will take longitudinal measurements, balance the gender of the participants, include people who are not married but do live together or people who live together even though they are divorced, and gather data from couples will help to eliminate these deficiencies.

In this study, the short version of the PN-RQ scale included different adjectives from the original study. This can be explained by cultural differences. Although characteristics in the evaluation of relationships that pertained more to Turkish participants were similar to US participants’ characteristics, they did not seem to coincide. Within the scope of this study, the short version of the PN-RQ scale is based on a sample of married couples. When considering Turkey’s cultural characteristics, the short version that can be used for romantic partners who are not married can be determined by means of future studies.

## 5. Conclusions

The measures for relationship quality are important both for understanding the nature of the relationships and for organizing intervention programs that will help nurture positive relationships. The PN-RQ scale is a practical measurement tool and suitable for use in scientific research and/or intervention studies. As there is evidence for reliability and validity in the Turkish version of the PN-RQ scale (see Appendix A) to measure both positive and negative aspects of the relationship, this scale will be highly functional for social and clinical psychologists who work on close relationship issues in Turkey. Additionally, this study is valuable due to its bifactorial structure, and cross-cultural studies can be performed using the Turkish version of the PN-RQ scale.

## Figures and Tables

**Table 1:** Descriptive Statistics and Exploratory Factor Loadings for the student sample.

Items	Mean (SD)	Factor	Items	Mean (SD)	Factor
Enjoyable	4.88 (1.12)	0.81	Miserable	1.31 (0.66)	0.66
Pleasant	5.02 (1.08)	0.86	Bad	1.51 (0.85)	0.78
Strong	4.98 (1.30)	0.62	Empty	1.61 (1.03)	0.79
Alive	4.65 (1.28)	0.88	Lifeless	1.77 (1.07)	0.76
Fun	4.91 (1.19)	0.85	Unpleasant	1.58 (0.95)	0.84
Full	4.48 (1.35)	0.80	Dull	1.72 (1.04)	0.72
Energizing	4.67 (1.34)	0.83	Weak	1.49 (0.91)	0.70
Exciting	4.77 ( 1.41)	0.81	Discouraging	1.82 (1.22)	0.70

**Table 2:** Descriptive Statistics and Exploratory Factor Loadings for the married sample.

Items	Mean (SD)	Factor	Items	Mean (SD)	Factor
Enjoyable	4.34 (1.31)	0.86	Miserable	1.83 (1.26)	0.91
Pleasant	4.26 (1.30)	0.88	Bad	1.83 ( 1.26)	0.98
Strong	4.55 (1.37)	0.57	Empty	1.86 (1.31)	0.91
Alive	3.89 (1.41)	0.90	Lifeless	2.22 (1.39)	0.78
Fun	3.92 (1.42)	0.91	Unpleasant	2.00 (1.37)	0.91
Full	3.75 (1.41)	0.90	Dull	2.10 ( 1.32)	0.79
Energizing	3.69 (1.46)	0.91	Weak	1.96 (1.38)	0.84
Exciting	3.60 ( 1.45)	0.93	Discouraging	2.20 (1.45)	0.84

**Table 3:** Fit Indices for Confirmatory Factor Analysis Models.

Models	χ^2^/*df*	RMSEA ^a^	CFI ^b^	NFI ^c^
Model 1: Correlated two-factor	7.08	0.12	0.98	0.97
Model 2: Orthogonal two-factor	9.52	0.13	0.97	0.96
Model 3: One-factor	31.66	0.37	0.88	0.88
Model 4: Bi-factor	4.70	0.09	0.99	0.98

Note: ^a^ RMSEA = root mean square error of approximation, ^b^ CFI = comparative fit index, ^c^ NFI = normed-fit index.

**Table 4:** Mean and Standard Deviations of Sub-Groups.

Satisfied n = 165	Dissatisfied n = 177	Indifferent n = 55	Ambivalent n = 56
112.66 (16.24)	74.45 (22.42)	98.48 (12.17)	99.28 (16.37)

## Appendix A. Turkish Version of the PN-RQ Scale

Lütfen, eşinizle olan ilişkinin sadece pozitif niteliklerini düşünüp, negatif niteliklerini göz ardı ederek, ilişkinizi aşağıdaki sıfatlarla değerlendiriniz.

**EŞİM İLE İLİŞKİM…**

**Table:**

	Hiç Doğru Değil	Çok az Doğru	Biraz Doğru	Oldukça Doğru	Çok Doğru	Bütünüyle Doğru
Zevkli	O	O	O	O	O	O
Keyifli	O	O	O	O	O	O
Güçlü	O	O	O	O	O	O
Canlı	O	O	O	O	O	O
Eğlenceli *	O	O	O	O	O	O
Dopdolu *	O	O	O	O	O	O
Enerji verici *	O	O	O	O	O	O
Heyecan verici *	O	O	O	O	O	O

* Items that compose the short version of the scale.

Lütfen, eşinizle olan ilişkinin sadece negatif niteliklerini düşünüp, pozitif niteliklerini göz ardı ederek, ilişkinizi aşağıdaki sıfatlarla değerlendiriniz.

**EŞİM İLE İLİŞKİM…**

**Table:**

	Hiç Doğru Değil	Çok az Doğru	Biraz Doğru	Oldukça Doğru	Çok Doğru	Bütünüyle Doğru
Berbat *	O	O	O	O	O	O
Kötü *	O	O	O	O	O	O
Boş *	O	O	O	O	O	O
Cansız	O	O	O	O	O	O
Tatsız *	O	O	O	O	O	O
Sıkıcı	O	O	O	O	O	O
Zayıf	O	O	O	O	O	O
Heves kırıcı	O	O	O	O	O	O

* Items that compose the short version of the scale.
